# Genome-Wide Characterization of TAZ Binding Sites in Mammary Epithelial Cells

**DOI:** 10.3390/cancers15194713

**Published:** 2023-09-25

**Authors:** Tao Liu, Jiaojiao Zhou, Yanmin Chen, Jia Fang, Song Liu, Costa Frangou, Hai Wang, Jianmin Zhang

**Affiliations:** 1 Department of Biostatistics and Bioinformatics, Roswell Park Comprehensive Cancer Center, Elm Street, Buffalo, NY 14203, USA; tao.liu@roswellpark.org (T.L.); jiaojiao.zhou@roswellpark.org (J.Z.); song.liu@roswellpark.org (S.L.); 2 Department of Cancer Genetics & Genomics, Roswell Park Comprehensive Cancer Center, Elm Street, Buffalo, NY 14203, USA; yanmin.chen@roswellpark.org; 3 Department of Pharmacology and Therapeutics, Roswell Park Comprehensive Cancer Center, Elm Street, Buffalo, NY 14203, USA; jia.fang@roswellpark.org; 4 Department of Molecular and Cellular Biology, Roswell Park Comprehensive Cancer Center, Elm Street, Buffalo, NY 14203, USA; costa.frangou@roswellpark.org (C.F.); hai.wang@roswellpark.org (H.W.)

**Keywords:** TAZ, breast cancer, TEAD, FOSL2, ChIP-seq, enhancer, ATAC-seq

## Abstract

**Simple Summary:**

The protein TAZ plays an important role in the Hippo signaling pathway that regulates important biological processes such as cell proliferation, controlled cell death, cancer stem cell traits, tumorigenesis, and resistance to therapies. Specifically, TAZ functions as a transcription coactivator that influences the transcription of various genes. Despite the importance of TAZ, genome-wide occupancy sites for TAZ remain poorly defined. Here, a tetracycline (tet)-inducible gene expression system was used to turn on TAZ activation in mammary epithelial cells, and we characterized genome-wide binding sites at different TAZ activation time points. We found that most TAZ binds to distant enhancer and non-coding regions of genes at earlier TAZ activation time points and binding shifts to nearby promoter regions of genes at later TAZ activation time points. We also found that TAZ activation results in chromatin architecture alterations. These results could lead to the identification of new therapeutic targets for breast cancer.

**Abstract:**

The transcriptional co-activator with PDZ binding motif (TAZ) is a key effector of the Hippo signaling pathway. We and others previously reported that high expression levels of TAZ are positively associated with decreased survival rates and shorter times to relapse in basal-like breast cancer (BLBC) patients. The oncogenic activity of TAZ involves the regulation of diverse signal transduction pathways that direct processes such as cell proliferation, migration, and resistance to apoptosis, albeit through poorly characterized gene expression programs. Here, using a tet-inducible system in mammary epithelial MCF10A cells, we have characterized the TAZ-regulated transcription program using RNA sequencing in a temporal and spatial manner. We further identified global TAZ binding sites at different TAZ activation time points by chromatin immunoprecipitation (ChIP) sequencing analysis. We found that the vast majority of TAZ was rapidly localized in enhancer regions at the early TAZ activation time point and then gradually spread to promoter regions. TAZ bound to enhancer regions following a switch in potential TEAD and FOSL2 transcription factor motifs. Furthermore, the ATAC sequencing analysis indicated that TAZ activation led to chromatin structural alterations. Together, our results have revealed the landscape of genome-wide TAZ binding sites and may lead to improvements in the current understanding of how TAZ regulates the gene expression program that contributes to the development of breast cancer.

## 1. Introduction

YAP/TAZ are transcriptional co-activators that serve as key components of the Hippo signaling pathway [1,2,3], which is essential for the regulation of cell proliferation, apoptosis, and organ size. Elevated expression levels of gene signatures for YAP/TAZ activity have been correlated with a high histological grade, enrichment of stem cell signatures, metastatic proclivity, and poor outcomes in breast cancer (BC) patient datasets [4,5,6,7,8]. Of particular note, TAZ mRNA and protein expression are markedly higher in triple-negative breast cancer (TNBC) than in the other BC subtypes [5,7,9]. We have also found that TAZ activation induces the epithelial-to-mesenchymal transition (EMT) and increases the cell proliferation and migration potential in BC cells [5,10]. These phenotypes are mainly mediated through interactions between TAZ and TEAD transcription factors [5,11]. Despite the importance of TAZ, genome-wide occupancy sites for TAZ remain poorly defined. Thus, further research into TAZ could lead to new therapeutic targets for aggressive forms of breast cancer.

TAZ-driven connective tissue growth factor (CTGF) and cysteine-rich angiogenic inducer 61 (CYR61) expression have been linked to taxol resistance in TNBC [12]. Because programmed death-ligand 1 (PD-L1)—an immunoregulatory protein—has been identified as a direct target of TAZ in human breast cancer cells, TAZ activation may play an important role in immune evasion [13]. The knockdown of both YAP and TAZ in TNBC MDA-MB-231 cells allowed for the identification of a large fraction of YAP/TAZ targets linked to processes related to cell cycle progression; these targets may contribute to cell cycle progression through Myc oncogene activation [14]. We have previously demonstrated that TAZ activates the epidermal growth factor receptor (EGFR) ligand amphiregulin, which induces growth factor-independent cell proliferation [15]. Genome-wide YAP/TAZ chromatin immunoprecipitation followed by sequencing (ChIP-seq) in MDA-MB-231 cells has shown that the majority of YAP/TAZ binding peaks are localized in distal enhancer regions [14]. Furthermore, activator protein 1 (AP-1) and the signal transducer and activator of transcription 3 (STAT3) transcription factors have been recently identified in concert with the TEAD transcription factor as being important drivers of the YAP/TAZ transcription program in breast cancer [14,16,17].

Here, using a tet-inducible system, the expression of TAZ-4SA (constitutively active TAZ form) was activated in TAZ-transduced MCF10A cells at different time points (24 h, 48 h, and 72 h) by doxycycline (Dox) administration. The TAZ-driven transcription program was evaluated using RNA sequencing (RNA-seq). We classified the components of the dynamic TAZ-driven transcription program into four groups, namely early upregulated, late upregulated, early downregulated, and late downregulated. Through the TAZ ChIP-seq analysis in parallel with transcriptome profiling, we revealed the landscape of genome-wide TAZ bind sites in response to TAZ activation both temporally and spatially. Furthermore, we found that TAZ activation induced alterations in the chromatin architecture, according to an Assay for Transposase-Accessible Chromatin using sequencing (ATAC-seq) analysis. The identified TAZ-regulated gene expression and TAZ binding peaks can help us to better understand the functional role TAZ plays in breast cancer.

## 2. Materials and Methods

### 2.1. Cell Culture

Tet-inducible, activated-TAZ-4SA-transduced, non-transformed human mammary epithelial MCF10A cells have been described previously [18]. Briefly, MCF10A cells were cultivated in a DMEM/F12 medium supply with 100 µg/mL streptomycin, 0.5 µg/mL hydrocortisone, 10 µg/mL insulin, 5% horse serum (HS), 100 ng/mL cholera toxin, 20 ng/mL epidermal growth factor (EGF), and 100 units/mL penicillin. The cells were cultured in a humidified atmosphere of 95% air and 5% CO_2_ at 37 °C. Doxycycline at a concentration of 0.05 µg/mL was used to treat the cells in order to induce TAZ activation at various time points (0 h, 24 h, 48 h, and 72 h).

### 2.2. Western Blots and Antibodies

The cell lysates were collected with RIPA buffer (50 mM Tris-pH 7.4, 150 mM NaCl, 1% NP-40, 0.5% Na deoxycholate, and 0.1% SDS), and 1× protease and phosphatase inhibitors (Halt Protease and Phosphatase Inhibitor Cocktail, Thermo Scientific^TM^; Grand Island, NE, USA) were added before the lysate collection. Briefly, cell lysates (30 µg) were loaded onto a 10% SDS-PAGE gel, and electrophoresis was performed. Proteins were transferred to PVDF (polyvinylidene difluoride) membranes for over 2 h at room temperature. Then, the PVDF membranes were blocked with 5% nonfat milk in TBST (Tris-buffered saline–0.1% Tween 20) for 1 h at room temperature. The membrane was incubated with a specific primary antibody at 4 °C overnight. Next, the membranes were washed 3 times with TBST buffer and then incubated with either a horseradish peroxidase (HRP) conjugated anti-rabbit antibody or mouse secondary antibody (Bio-Rad; Hercules, CA, USA) for 1 h at room temperature. The membranes were washed 3 times with TBST. The enhanced chemiluminescence (ECL) plus Western blotting detection reagents were added accordingly to detect protein bands. Flag (M2) antibody was purchased from Sigma-Aldrich (Saint Louis, MO, USA); glyceraldehyde-3-phosphate dehydrogenase (GAPDH) antibody was purchased from Ubiquitin-Proteasome Biotechnologies (Dallas, TX, USA); TAZ antibody was purchased from Cell Signaling Technology (Danvers, MA, USA).

### 2.3. RNA Extraction and RNA-Seq

The tet-inducible, TAZ-4SA-transduced MCF10A cells were treated with 0.05 µg/mL Dox for 24 h, 48 h, and 72 h. Total RNA was extracted by using Trizol Reagent (Life Technologies; Carlsbad, CA, USA) according to the manufacturer’s protocol. RNAs were further processed for next-generation sequencing at Roswell Park’s genomic core facility. Briefly, the sequencing libraries were prepared with the mRNA HyperPrep kit (Roche; Basel, Switzerland) from 500 ng total RNA. Following the manufacturer’s instructions, the first step was selected for PolyA RNA using the mRNA Capture Beads (KAPA BIOSYSTEMS; Wilmington, NC, USA). After PolyA selection, the remaining RNA was purified, fragmented, and primed for cDNA synthesis. Fragmented RNA was then reverse-transcribed into first-strand cDNA using random primers. The next step removed the RNA template and synthesized a replacement strand, incorporating dUTP in place of dTTP to generate double-strand cDNA (ds-cDNA). Pure Beads (Roche; Basel, Switzerland) were used to separate the ds-cDNA from the second strand reaction mix, resulting in blunt-ended cDNA. A single “A” nucleotide was then added to the 3′ ends of the blunt fragments. Multiple indexing adapters, containing a single “T” nucleotide on the 3′ end of the adapter, were ligated to the ends of the ds-cDNA, thereby preparing these samples for hybridization onto a flow cell. Adapter ligated libraries were amplified by PCR, purified using Pure Beads, and validated for an appropriate size on a 4200 TapeStation D1000 Screentape (Agilent Technologies, Inc.; Santa Clara, CA, USA). The DNA libraries were quantitated using KAPA Biosystems qPCR kit (Wilmington, NC, USA), and DNA was pooled together in an equimolar fashion following the experimental design criteria. Each pool was denatured and diluted to 200 pM with 1% PhiX control library added. The resulting pools were then loaded into a NovaSeq 6000 (Illumina Inc.; San Diego, CA, USA) flow cell for 100 paired end (PE) sequencing. Sequencing was performed on a NovaSeq6000 following the manufacturer’s recommended protocol (Illumina Inc.; San Diego, CA, USA) using the NovaSeq 200 cycle Reagent cartridge. On average, 50 million PE reads were generated.

### 2.4. RNA-Seq Analysis

We mapped the RNA-seq PE reads to the human transcriptome using STAR [19] based on the human genome assembly GRCh38 and GENCODE gene annotation Version 22. The STAR algorithm applies soft-clip during alignment, and it generates two types of alignments: one based on the coordination on the transcriptome (for each transcript) and the other based on the genomic coordination. We used the RSEM algorithm [20] to estimate the transcript counts (expected count values) for each known gene in GENCODE v22 based on the alignment to the transcriptome. The count data were further loaded using the tximport tool in R and analyzed by DESeq2 [21]. To perform a time-course analysis, we used the Likelihood Ratio Test (LRT) provided by DESeq2 and identified 3012 differentially expressed genes (DEGs) over all time points, with a false discovery rate (FDR) cutoff of 0.05 and minimum absolute log2 fold change of 1. Then, we used the degReport package version 1.30.0 (http://lpantano.github.io/DEGreport/ accessed on 6 November 2021) in R to identify eight different expression change patterns over time among the 3012 genes, and we manually grouped those patterns into four categories: early up, early down, late up, and late down. To compare the DEGs in TAZ-transduced MCF10A cells from the No-Dox treatment, the DEGs of each of the four timepoints—Dox treatment for 24 h, 48 h, and 72 h—were called separately using DESeq2 (version 1.34.0), with an FDR cutoff of 0.05 and minimum absolute log2 fold change of 1. A KEGG (Kyoto Encyclopedia of Genes and Genomes) Pathway analysis on gene sets was performed using the clusterProfiler package (version 4.2.0) [22] in Bioconductor.

### 2.5. ChIP Assays and ChIP-Seq

We performed ChIP assays using the SimpleChIP^®^ Plus Sonication Chromatin IP Kit (Cell Signaling Technologies; Danvers, MA, USA) according to the manufacturer’s instructions. Briefly, TAZ-transduced MCF10A cells were treated without or with 0.05 µg/mL Dox for 24 h, 48 h, or 72 h. The cells were cross-linked with 1% formaldehyde for 10 min at room temperature, and the reaction was stopped by adding glycine (final concentration of 0.1 M); cross-linked cells were collected in ice-cold phosphate-buffered saline (PBS) and washed twice with PBS. Next, the cell pellets were resuspended in sonication cell lysis buffer and sonicated in sonication nuclear lysis buffer on ice using a 30 s on/off cycle condition for 10 min. The size of the DNA fragments (500–800 bp) was confirmed by agarose electrophoresis. The immunoprecipitation was performed using 5 µg of antibody to TAZ (Cell Signaling Technologies, #4883; Danvers, MA, USA) or the IgG control (Cell Signaling Technologies, #2729; Danvers, MA, USA). ChIPed DNAs were further processed for next-generation sequencing at the Roswell Park genomic core facility. Briefly, 2 ng of ChIPed DNA was used to generate a library for next-generation sequencing using ThruPLEX DNA seq kits (Takara Bio, Inc.; Boston, MA, USA), as per the manufacturer’s instructions. ThruPLEX uses stem–loop adapters to construct high-quality libraries in a fast and efficient manner. In the first step, template preparation, the DNA was repaired, and this yielded molecules with blunt ends. Next, stem–loop adaptors with blocked 5′ ends were ligated with a high efficiency to the 5′ end of the genomic DNA, while leaving a nick at the 3′ end. In the final step, the 3′ ends of the genomic DNA were extended to complete library synthesis, and Illumina-compatible indexes were added through a high-fidelity amplification. The enriched library was quantitated using quantitative PCR (KAPA Biosystems; Wilmington, NC, USA), normalized, and pooled. Each pool was denatured and diluted to 200 pM with 1% PhiX control library added. The resulting pools were then loaded into the appropriate NovaSeq flow cell for 100 PE sequencing. Sequencing was performed on a NovaSeq6000 following the manufacturer’s recommended protocol (Illumina Inc.; San Diego, CA, USA) using the 300 cycle NovaSeq Reagent cartridge. On average, 50 million PE reads were generated.

### 2.6. ChIP-Seq Data Analysis

The sequenced raw reads were subjected to quality control (QC) procedures, which involved the trimming of bases with low-quality scores prior to read mapping. We used FastQC to scan base quality scores. The filtered reads were mapped against the latest reference genome hg38 using the Burrows–Wheeler Aligner (BWA) [23]. After mapping, we followed the ChIP-Seq analysis guideline [24] to detect technical artifacts or sequencing failures, which involved an assessment of the normalized and relative strand cross-correlation (NSC and RSC) values suggested by the ENCODE consortium and immunoprecipitation (IP) strength according to the FRiP (fraction of reads in peaks) scores. Our dataset’s overall QC measurements were compared with all the publicly available ChIP-Seq datasets collected in CistromeDB [25]. We then used MACS2 [26] to identify significant peaks for all samples from stimulated and unstimulated cells with an FDR cutoff of 0.01. After peak calling, the results were evaluated for overlap with known DNA hypersensitive (DHS) regions merged from different cell types (collected by the ChiLin pipeline [27]) and known blacklist regions [28], where artificial ChIP enrichment would be expected. Higher overlap with DHS regions and lower overlap with blacklist regions is a sign of high-quality data. The conservation across vertebrates and primates at the peak regions and the enrichment of peaks at the promoter, exons, introns, and intergenic regions were computed to evaluate the quality of the experiments. All the above tools were implemented in ChiLin [27], which allowed us to conveniently process and evaluate our datasets in a uniform way. After we obtained the TAZ binding sites for each condition, we utilized the GIGGLE [29] tool to search for the enrichment of the known transcriptional regulator ChIP-seq peaks, including 1314 regulators collected in the Cistrome Database (accessed on 6 November 2021) [25]. We analyzed peak enrichment over genome features using the ChIPSeeker tool (version 1.30.0) [30] in Bioconductor (version 3.14.0). The DNA binding motifs at TAZ binding sites were analyzed using HOMER [31].

### 2.7. ATAC-Seq Sample Preparation and ATAC-Seq

The ATAC-seq sample process and library preparation were performed as described [32]. Briefly, inducible TAZ-4SA-transduced MCF10A cells were treated without or with Dox for 72 h. Then, 50,000 cells were harvested, and transposition reactions were performed. Following transposition, DNA was purified using a Qiagen MinElute PCR Purifi-cation Kit (Venlo, The Netherlands) and PCR-amplified for 10 cycles. The quality of ATAC-seq libraries was qualitatively assessed using Bioanalyzer, and next-generation sequencing was performed by Roswell Park’s genomic core facility. The amplified products were purified with AmpureXP beads (Beckman Coulter; Brea, CA, USA), followed by verification of the final library profiles by the HSD1000 screentape on a Tapestation 4200 (Agilent Technologies Inc.; Santa Clara, CA, USA). The libraries were quantitated using KAPA Biosystems qPCR kits (Roche; Basel, Switzerland) and pooled together in an equimolar fashion following experimental design criteria. The resulting pool was then loaded into the appropriate NovaSeq Reagent cartridge for 100 PE sequencing and sequenced on a NovaSeq6000 following the manufacturer’s recommended protocol (Illumina Inc.; San Diego, CA, USA). On average, 100 million PE reads were generated.

### 2.8. ATAC-Seq Data Analysis

Our pipeline for ATAC-seq analysis began with aligning the ATAC-seq PE sequencing reads to the human reference genome hg38 using aligner BWA, followed by the identification of accessible regions or “peaks” by MACS2 with an FDR cutoff of 0.05. Two biological replicates for each condition—No Dox or Dox—were pooled. The pipeline code can be found at https://github.com/macs3-project/genomics-analysis-pipelines (accessed on 7 November 2021). We evaluated data quality by checking the mapping ratio, percentage of reads mapped to mitochondria, and percentage of reads mapped to gene promoter regions. We also evaluated the peak calling quality by comparing so-called chromatin accessible regions with known DHS regions merged from different cell types and ENCODE blacklist regions.

To identify the differentially accessible regions (DARs) between Dox and No-Dox conditions, we first merged the peaks called from both conditions to obtain the union space of potential accessible regions under either condition, and then we divided the regions into 100 bps bins. For each bin, we counted the number of reads in each of the four samples and made the count table in bins. The table was later loaded into DESeq2 to call the DARs with an FDR cutoff of 0.1 and absolute fold change of 1.2. We utilized the GIGGLE tool to search for the enrichment of known transcriptional regulators based on CistromeDB in identified DARs.

### 2.9. Statistical Analyses

All statistical and bioinformatics analyses were performed using R (version 4.1.1) and the Bioconductor software suite (version 3.14.0).

## 3. Results

### 3.1. Assessment of Tet-Inducible TAZ Expression in MCF10A Cells

**Figure 1:**
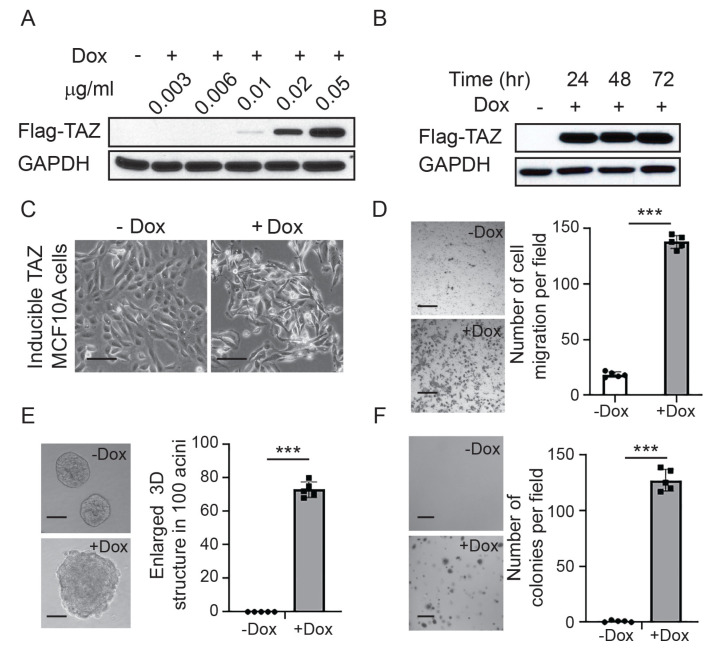
Characterization of the inducible TAZ activation system. (**A**) Immunoblot detection of exogenous TAZ expression in response to different does of doxycycline (Dox). GAPDH was used as the loading control. (**B**) Immunoblot detection of exogenous TAZ expression in response to 0.05 µg/mL doxycycline at different time points. GAPDH was used as the loading control. (**C**) TAZ activation induced cell morphological changes from curbstone epithelial cells to fibroblast-like mesenchymal cells. Scale bar = 100 µm (**D**) Representative images and quantification of cell migration under the condition without or with Dox. TAZ activation increased cell migration. Scale bar = 200µm (**E**) Representative images and quantification of three-dimensional (3D) acini formation under the condition without or with Dox. TAZ activation led to enlarged 3D culture acini formation. Scale bar = 100µm (**F**) Representative images and quantification of colonies in soft agar under the condition without or with Dox treatment. TAZ activation induced colony formation in soft agar. Scale bar = 250 µm Data are shown as the mean ± standard deviation (SD). Statistical analyses were run with unpaired two-tailed Student’s *t*-tests; *** *p* < 0.001. The uncropped bolts are shown in Supplementary Materials.

To optimize the tet-inducible TAZ activation, we treated the TAZ-transduced MCF10A cells with different doses of Dox. We found that exogenous TAZ expression can be activated at a minimum of 0.02 µg/mL Dox administration (Figure 1A). TAZ protein levels were stably expressed in response to 0.05 µg/mL Dox treatment after 24 h (Figure 1B). Consistent with earlier findings, TAZ activation dramatically induced cell morphological changes from curbstone epithelial cells to fibroblast-like mesenchymal cells, which indicates that TAZ activation can induce the EMT (Figure 1C). Furthermore, TAZ activation led to significant increases in cell migration according to Boyden chamber cell migration assays (Figure 1D). We further performed 3D organoid culture assays, and we found that TAZ activation induced massive enlarged acini formation, which indicates that TAZ activation can drive cellular hyper-proliferation (Figure 1E). Finally, we confirmed that TAZ activation can lead to cellular transformation using colony formation assays in soft agar (Figure 1F). Collectively, the results demonstrate that this is a robust tet-inducible TAZ activation system in MCF10A cells.

### 3.2. Time-Course Characterization of the TAZ-Driven Transcription Program

**Figure 2:**
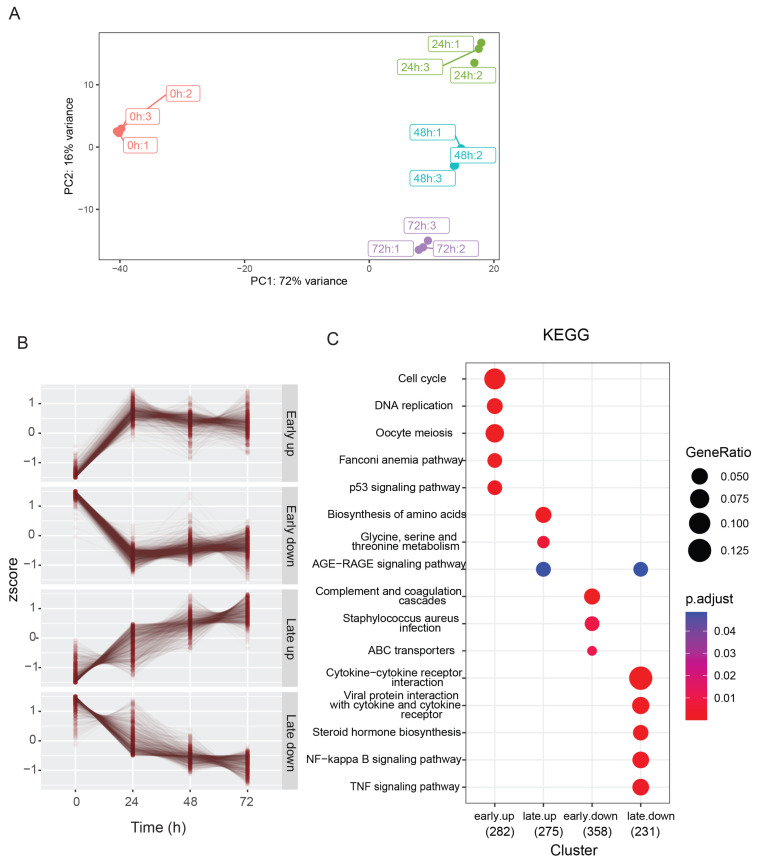
TAZ activates distinctive transcription programs. (**A**) Principal component analysis (PCA) plot of the TAZ-driven transcriptome in response to Dox treatment at different time points. (**B**) Four gene expression patterns were observed after Dox administration at different time points. (**C**) KEGG analysis identified the pathway alterations in response to TAZ activation. GeneRatio was calculated as the ratio of the number of genes within the KEGG pathway versus the total number of genes in the four gene sets (as shown in the x-axis). The p.adjust value represents the multiple comparison adjusted *p*-value derived from the Benjamini–Horchberg method.

To characterize the TAZ-driven transcription program in a time-dependent manner, we harvested RNA from the cells without Dox or with Dox treatment at 24 h, 48 h, and 72 h and performed RNA-seq analysis. The principal component analysis (PCA) plot revealed a distinct transcription program in response to Dox treatment with high reproducibility (Figure 2A). The RNA-seq analysis identified 3012 significant DEGs over three time points after TAZ activation in comparison with data from the treatment without Dox. We classified the upregulated and downregulated genes into four categories: (1) early upregulated genes (*n* = 282); (2) early downregulated genes (*n* = 358); (3) late upregulated genes (*n* = 275); (4) late downregulated genes (*n* = 231) (Figure 2B; Supplementary Table S1). The KEGG analysis indicated that the cell cycle and cell proliferation genes were in the early upregulated gene category, whereas the amino-acid biosynthesis genes were in the late upregulated gene category (Figure 2C). Interestingly, we found that TAZ activation suppressed complement components and the NF kappa B and tumor necrosis factor (TNF) signaling pathways (Figure 2C). These data indicate that TAZ activation regulates diverse biological processes.

### 3.3. Landscape of the Genome-Wide TAZ Binding Sites

**Figure 3:**
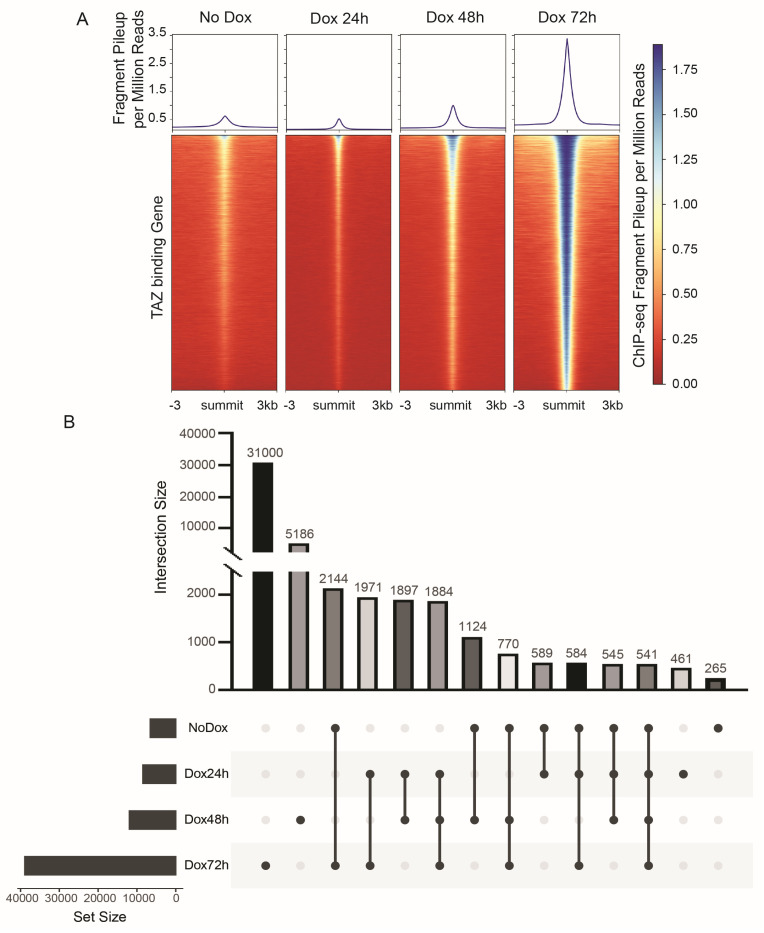
Genome-wide characterization of TAZ binding sites. (**A**) Representative aggregation plot and heatmap of ChIP-seq signals around genome-wide TAZ binding peaks. The values are the fragment pileup per million reads from MACS2. Please note that the heatmap color saturates at 1.8 as the darkest blue; the actual value can be higher than 1.8. (**B**) The UpSet plot shows overlap or distinctive TAZ binding peaks at different time points after Dox administration. Each column shows the number of binding sites distinctive to each category (single dot) or common among categories (connected dots).

To reveal global TAZ binding sites, we carried out a ChIP-Seq analysis using an anti-TAZ antibody under the without-Dox condition or at 24 h, 48 h, and 72 h after Dox administration (Figure 3A). Among these different TAZ activation time points, we identified about 5000 commonly gained TAZ binding peaks at 24 h through 72 h after TAZ activation (Figure 3B). We also found 461 distinct TAZ binding peaks at 24 h, 5186 peaks at 48 h, and 31,000 peaks at 72 h (Figure 3B).

**Figure 4:**
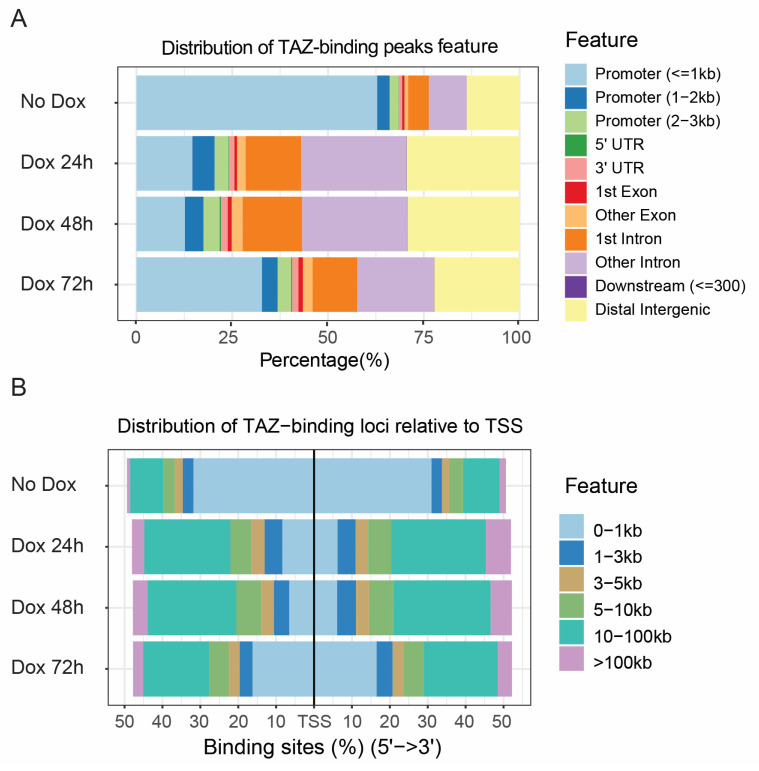
Genome-wide distribution of TAZ binding peaks in response to Dox treatment. (**A***)* Distribution of TAZ binding peak features at different TAZ activation time points. (**B**) Distribution of TAZ binding peaks relative to transcription factor start sites (TSSs).

We used ChIPSeeker (version 1.30.0) [30] to determine the enriched genomic features at TAZ binding sites. Interestingly, we found that more than 70% of TAZ was bound to promoter regions (0–3 kb) under normal growth conditions in MCF10A cells (Figure 4A). However, under the TAZ activation condition, we found that approximately 75% of TAZ binding peaks were localized in other intron or distal intergenic regions, and less than 25% of TAZ binding peaks were localized in the promoter regions after 24 h of Dox treatment (Figure 4A). In contrast, we found that about 40% of TAZ binding peaks were localized in the promoter regions, and about 52% of TAZ binding peaks were localized in the other intron or distal intergenic regions after 72 h of Dox treatment (Figure 4A). Consistently, we revealed that approximately 25% of TAZ binding peaks were localized 0–3 kb away relative to the transcription starting site (TSS) from both strands, and the vast majority of TAZ binding peaks were localized in the 10–100 kb regions relative to the TSS at 24 h after TAZ activation (Figure 4B). At 72 h TAZ activation, the number of TAZ binding peaks increased to approximately 40% relative to the location adjacent to the TSS and decreased to 35% in 10–100 kb regions relative to the TSS (Figure 4B). These results suggest that TAZ dynamically binds to genomic regions.

### 3.4. Dynamic Alterations of PLSs, pELSs, and dELSs Containing TAZ Binding Peaks

To reveal patterns of TAZ binding to its targets, we analyzed several well-known TAZ targets over the study’s time course (Supplementary Figure S1). Interestingly, we detected three TAZ binding patterns: (1) TAZ binds to a promoter first and then binding extends to upstream enhances, such as with CTGF and CYR61; (2) TAZ simultaneously binds to upstream enhancers and promoters, such as with ANKRD1 and CCDC80C; (3) TAZ binds to a promoter and binding extends to the gene body, such as with KIBRA and AMOTL2.

**Figure 5:**
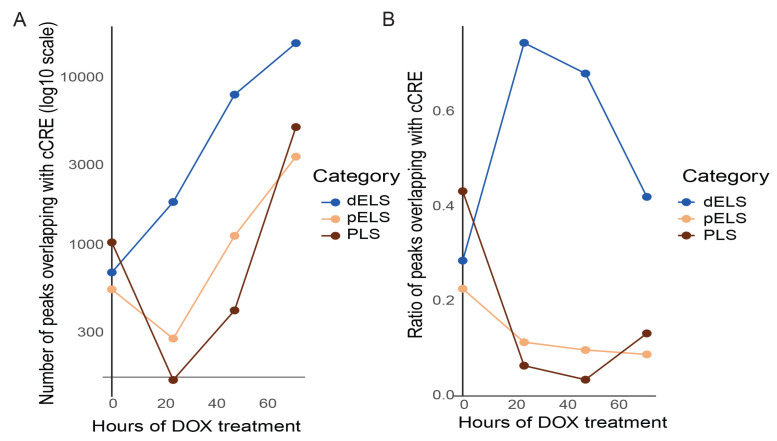
Dynamic alterations in promoter-like signatures (PLSs), proximal enhancer-like signatures (pELSs), and distal enhancer-like signatures (dELSs) containing TAZ binding peaks. (**A**) Numbers of TAZ binding peaks contain PLSs, pELSs, and dELSs at different TAZ activation time points. (**B**) Ratio of TAZ binding peaks overlapping with the cCRE (candidate cis-regulatory elements) dataset.

To understand the dynamics of TAZ binding to the distal enhancer, proximal enhancer, and promoter regions, we overlapped TAZ binding sites with ENCODE cCRE (candidate cis-regulatory elements) regions that contains over 1 million human regulatory DNA elements (Figure 5A). Under the normal growth condition, we found that TAZ binding peaks contained relatively equal numbers of promoter-like signatures (PLSs), proximal enhancer-like signatures (pELSs), and distal enhancer-like signatures (dELSs). However, in response to TAZ activation, dELSs containing TAZ binding peaks dramatically increased. In contrast, the PLSs and pELSs in TAZ binding peaks decreased significantly. These data were confirmed when we compared the ratio of PLSs, pELSs, and dELSs containing peaks at different TAZ activation time points (Figure 5B).

### 3.5. Analysis of Transcription Factor Motifs in TAZ Binding Peaks

**Figure 6:**
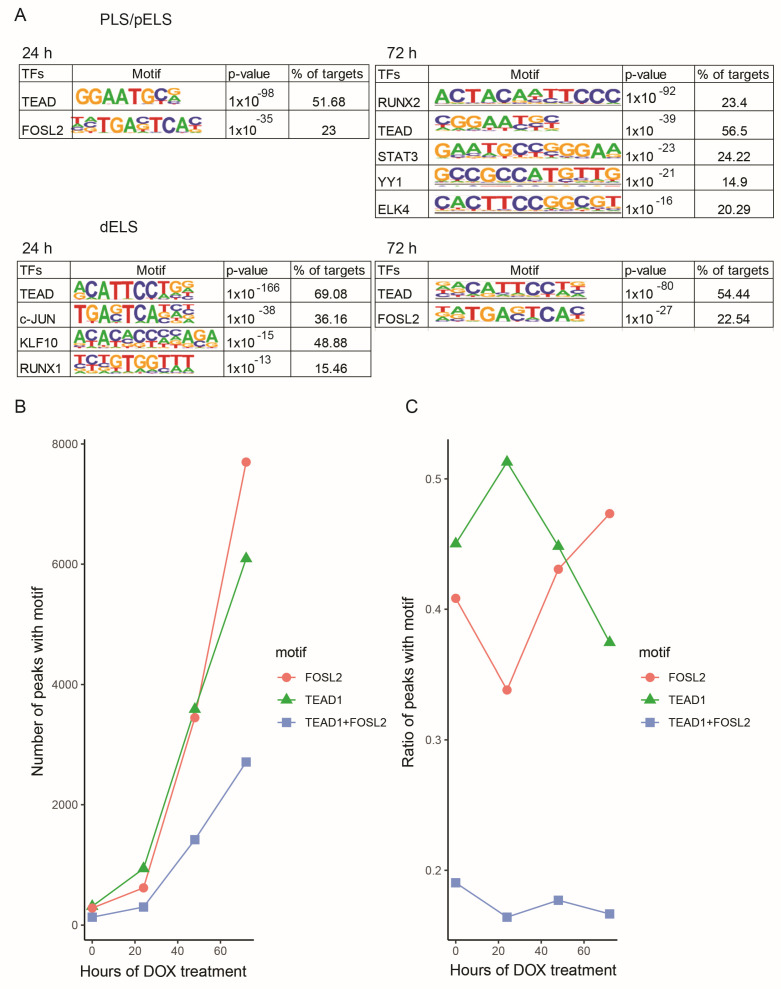
Analysis the transcription factor (TF) motifs in TAZ binding peaks. (**A**) Analysis of TF motifs in TAZ binding peaks after 24 h and 72 h during the Dox treatment. (**B**) Numbers of FOSL2 and TEAD1 and coexistence of TEAD1 and FOSL2 motifs in TAZ binding peaks containing dELS. (**C**) Ratio of FOSL2 or TEAD1 alone and coexistence of TEAD1 and FOSL2 in TAZ binding peaks.

To analyze the transcription factor (TF) motifs in TAZ binding peaks, we performed de novo motif analysis using HOMER. Then, the discovered motifs were compared with known motifs in the HOMER database. We found that the TEA domain factor (including TEAD1/2/3/4) and bZIP class factor (including JUNB, FOSL2, and BATF) motifs were the top two TF motifs in pELS/PLS regions 24 h after Dox treatment (Figure 6A). After 72 h of Dox treatment, we observed Runt domain factor (e.g., RUNX2), TEA domain factor (e.g., TEAD), STAT domain factor (e.g., STAT3), and YY1 and tryptophan cluster factor (e.g., ELK4) motifs in TAZ binding peaks.

We detected a switch in the TEAD and FOSL2 motifs at the early time point of TAZ activation (Figure 6B,C). The colocalization of TEAD/FOSL2 motifs remained at a steady status during TAZ activation (Figure 6C). Interestingly, we found that TAZ was directly bound to TEAD1, TEAD3, and TEAD4 promoter regions, but not TEAD2, which indicates that TAZ mainly activates TEAD1, TEAD3, and TEAD4 in mammary epithelial cells (Supplementary Figure S2A). We observed that TAZ was bound to FOSL1 and FOSL2 at the later TAZ activation time point (72 h) (Supplementary Figure S2B). These data indicate that TAZ activates distinct TEAD family members in mammary epithelial cells. Furthermore, TAZ binds to TEAD or FOSL2 transcription factors separately and may regulate different sets of gene expression.

### 3.6. TAZ Activation Alters Chromatin Architecture

**Figure 7:**
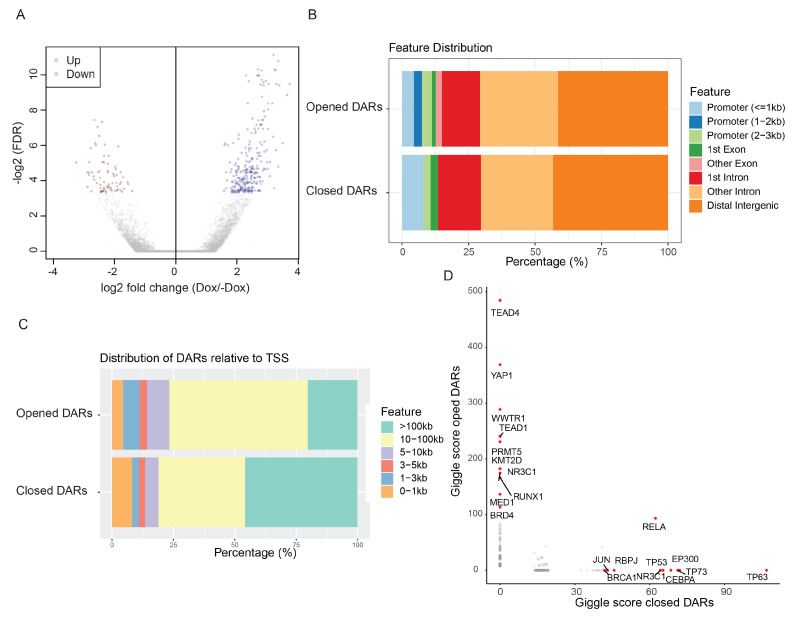
TAZ activation promotes the alterations of chromatin architecture. (**A**) Volcano plot of opened and closed differentially accessible regions (DARs). (**B**) Distribution of opened and closed DARs. (**C**) Distribution of opened and closed DARs relative to the transcription starting site (TSS). (**D**) Transcription factors in opened and closed DARs identified through the GIGGLE analysis.

Finally, to assess genome-wide chromatin accessibility in response to TAZ activation, we carried out the ATAC-seq analysis at 72 h after Dox administration. We found that TAZ activation led to both opened and closed DARs (Figure 7A). The vast majority of DARs in response to TAZ activation were widely localized in distal intergenic and other intron regions (Figure 7B). Less than 10% of opened or closed DARs were located in promoter regions (Figure 7B,C). Utilizing GIGGLE analysis to determine the potential transcription factor binding sites in the TAZ activation-induced DARs, we found that chromatin modifiers PRMT5, KMT2D, MED1, and BRD4 were highly associated with opened DARs (Figure 7D). These data indicate that TAZ activation drives chromatin architecture alterations and potentially acts through the recruitment of specific histone modifiers.

## 4. Discussion

TAZ is one of the major Hippo pathway transducers and plays an important role in organ size control, mechanotransduction, cell proliferation, cancer stem cell (CSC) traits, resistance to chemotherapy, and tumorigenesis [33,34,35,36]. Using a tet-inducible system, we have systematically characterized the landscape of genome-wide TAZ binding sites in mammary epithelial cells.

Consistent with a recent report, we found that the vast majority of TAZ binds to distal intergenic regions early on during TAZ activation (i.e., at 24 h) [14]. Interestingly, we revealed that TAZ binding peaks dramatically increased around TSS regions at a later TAZ activation time point (i.e., at 72 h). We found three distinctive TAZ binding patterns to its targets, which differed in regard to the type of binding site (e.g., promoter and enhancer) and temporal features of binding (e.g., early vs. late binding). These different binding patterns may result from the chromatin accessibility of its targets. Whether there are different transcription factor motifs in these binding sites warrants further analysis.

TAZ does not contain a DNA binding motif and functions as a transcriptional co-activator that is recruited by the TEAD family of transcription factors [37]. More recently, it has been shown that TAZ also binds to the transcription factors AP1 and STAT3 [14,16]. The previous working model was TAZ recruited by TEADs, and act together with AP-1 factors at genomic regions containing both TEAD and AP1 sequence motifs [14]. Our data align with recent findings that TAZ binds to TEAD or AP1 separately, i.e., only a minority of TAZ binds to the TEAD and AP1 motifs together. Furthermore, we found that TAZ was rapidly recruited to TEAD motif-containing sites and reduced binding to AP-1 motif containing sites in response to TAZ activation (24 h). At a later TAZ activation time point (72 h), TAZ was redistributed individually between TEAD and AP-1 motif-containing sites. These results indicate that TAZ may co-operate with TEAD and AP-1 separately to regulate the gene expression program. Interestingly, we also detected TAZ binding peaks in TEAD 1, 3, and 4 family members, as well as FOSL1 and FOSL2 promoter regions. This is consistent with a recent report that YAP/TAZ binds to the promoter region of FOS to stimulate its transcription [17].

Previously, it has been reported that YAP/TAZ can recruit the mediator complex to YAP-bound regulatory elements (YREs) [38]. Our ATAC-seq analysis indicates that TAZ activation induces alterations in the chromatin architecture and may involve the recruitment mediator MED1. Furthermore, we found that TAZ activation may result in the recruitment of histone modifiers, such as histone arginine methyltransferase 5 (PRMT5) and **histone**-lysine N-methyltransferase (KMT2D) to open DARs. Further functional analysis is warranted to precisely understand whether TAZ activation drives histone modification and consequently leads to epigenetic alterations.

## 5. Conclusions

In summary, through the use of a tet-inducible system, we dissected the TAZ-regulated transcription program and genome-wide TAZ binding sites in response to TAZ activation in mammary epithelial cells. Our studies will help us to further understand the biological function of TAZ during breast cancer development.

## Data Availability

The raw RNA-seq, ChIP-seq, and ATAC-seq data presented in the study were deposited in the Gene Expression Omnibus (GEO) repository.

## Supplementary Materials

The following supporting information can be downloaded at: https://www.mdpi.com/article/10.3390/cancers15194713/s1, Figure S1: Identification TAZ binding patterns for its targets; Figure S2: TAZ directly binds to TEAD and FOSL transcription factor promoters; Table S1: KEGG pathway analysis in response to TAZ activation.
