# Supplementary material for: Genome-Wide Characterization of TAZ Binding Sites in Mammary Epithelial Cells
Source: Cancers (Basel). 2023 Sep 25;15(19):4713. doi: 10.3390/cancers15194713 (PMC10571831; doi:10.3390/cancers15194713)
Supplement: Supplementary file 1 [file cancers-15-04713-s001.zip › cancers-2543387-supplementary.pdf]

Supplemental Figure legends

Figure S1 Identification TAZ binding patterns for its targets.

Representative images of TAZ binding peaks on genomic loci of CTGF and CYR61 (A); ANKRD1 and CCDC80 (B); KIBRA and AMOTL2 (C) in response to Dox treatment.

Figure S2 TAZ directly binds to TEAD and FOSL transcription factor promoters.

A. Representative images of TAZ binding peaks on promoter regions of TEAD1, 2 and 4, but not TEAD2.

B. Representative images of TAZ binding peaks on promoter and upstream enhancer regions of FOSL1 and FOSL2.

Supplemental Table S1: KEGG pathway analysis in response to TAZ activation.

	Cluster	GeneRatio	pvalue	p.adjust	qvalue	Count	Description
1	early.up	38/302	9.25E-22	2.40E-19	2.28E-19	38	Cell cycle - Homo sapiens (human)
2	early.up	13/302	1.30E-10	1.68E-08	1.60E-08	13	DNA replication - Homo sapiens (human)
3	early.up	27/302	8.76E-10	7.56E-08	7.19E-08	27	Motor proteins - Homo sapiens (human)
4	early.up	19/302	1.68E-07	1.09E-05	1.04E-05	19	Oocyte meiosis - Homo sapiens (human)
5	early.up	11/302	2.93E-06	0.000152	0.000144	11	Fanconi anemia pathway - Homo sapiens (human)
6	early.up	11/302	5.59E-05	0.002335	0.002221	11	p53 signaling pathway - Homo sapiens (human)
7	early.up	13/302	6.31E-05	0.002335	0.002221	13	Progesterone-mediated oocyte maturation - Homo sapiens (human)
8	early.up	8/302	8.18E-05	0.002649	0.002519	8	Homologous recombination - Homo sapiens (human)
9	early.up	6/302	0.000119	0.003412	0.003245	6	Mismatch repair - Homo sapiens (human)
10	early.up	15/302	0.000438	0.011347	0.010791	15	Cellular senescence - Homo sapiens (human)
11	early.up	7/302	0.000845	0.0199	0.018925	7	Base excision repair - Homo sapiens (human)
12	early.up	10/302	0.001105	0.023857	0.022688	10	Gap junction - Homo sapiens (human)
13	late.up	13/280	8.67E-07	0.000253	0.000242	13	Biosynthesis of amino acids - Homo sapiens (human)
14	late.up	8/280	3.97E-05	0.005797	0.005538	8	Glycine, serine and threonine metabolism - Homo sapiens (human)
15	late.up	11/280	0.000442	0.043019	0.041096	11	AGE-RAGE signaling pathway in diabetic complications - Homo sapiens (human)
16	early.down	17/365	1.11E-07	3.25E-05	3.16E-05	17	Complement and coagulation cascades - Homo sapiens (human)
17	early.down	14/365	5.72E-05	0.008381	0.00816	14	Staphylococcus aureus infection - Homo sapiens (human)
18	early.down	9/365	0.000104	0.010183	0.009914	9	ABC transporters - Homo sapiens (human)
19	late.down	29/233	2.24E-09	6.09E-07	5.61E-07	29	Cytokine-cytokine receptor interaction - Homo sapiens (human)
20	late.down	13/233	3.17E-06	0.000432	0.000398	13	Viral protein interaction with cytokine and cytokine receptor - Homo sapiens (human)
21	late.down	10/233	6.31E-06	0.000572	0.000527	10	Steroid hormone biosynthesis - Homo sapiens (human)
22	late.down	13/233	1.38E-05	0.000935	0.000861	13	TNF signaling pathway - Homo sapiens (human)
23	late.down	12/233	2.63E-05	0.00143	0.001317	12	NF-kappa B signaling pathway - Homo sapiens (human)
24	late.down	11/233	0.000107	0.004553	0.004194	11	Amoebiasis - Homo sapiens (human)
25	late.down	9/233	0.000117	0.004553	0.004194	9	Epithelial cell signaling in Helicobacter pylori infection - Homo sapiens (human)
26	late.down	10/233	0.000225	0.007424	0.006838	10	Rheumatoid arthritis - Homo sapiens (human)
27	late.down	10/233	0.000246	0.007424	0.006838	10	IL-17 signaling pathway - Homo sapiens (human)
28	late.down	9/233	0.001564	0.041509	0.038232	9	Hematopoietic cell lineage - Homo sapiens (human)
29	late.down	9/233	0.001679	0.041509	0.038232	9	AGE-RAGE signaling pathway in diabetic complications - Homo sapiens (human)
30	late.down	9/233	0.002205	0.049987	0.046041	9	C-type lectin receptor signaling pathway - Homo sapiens (human)

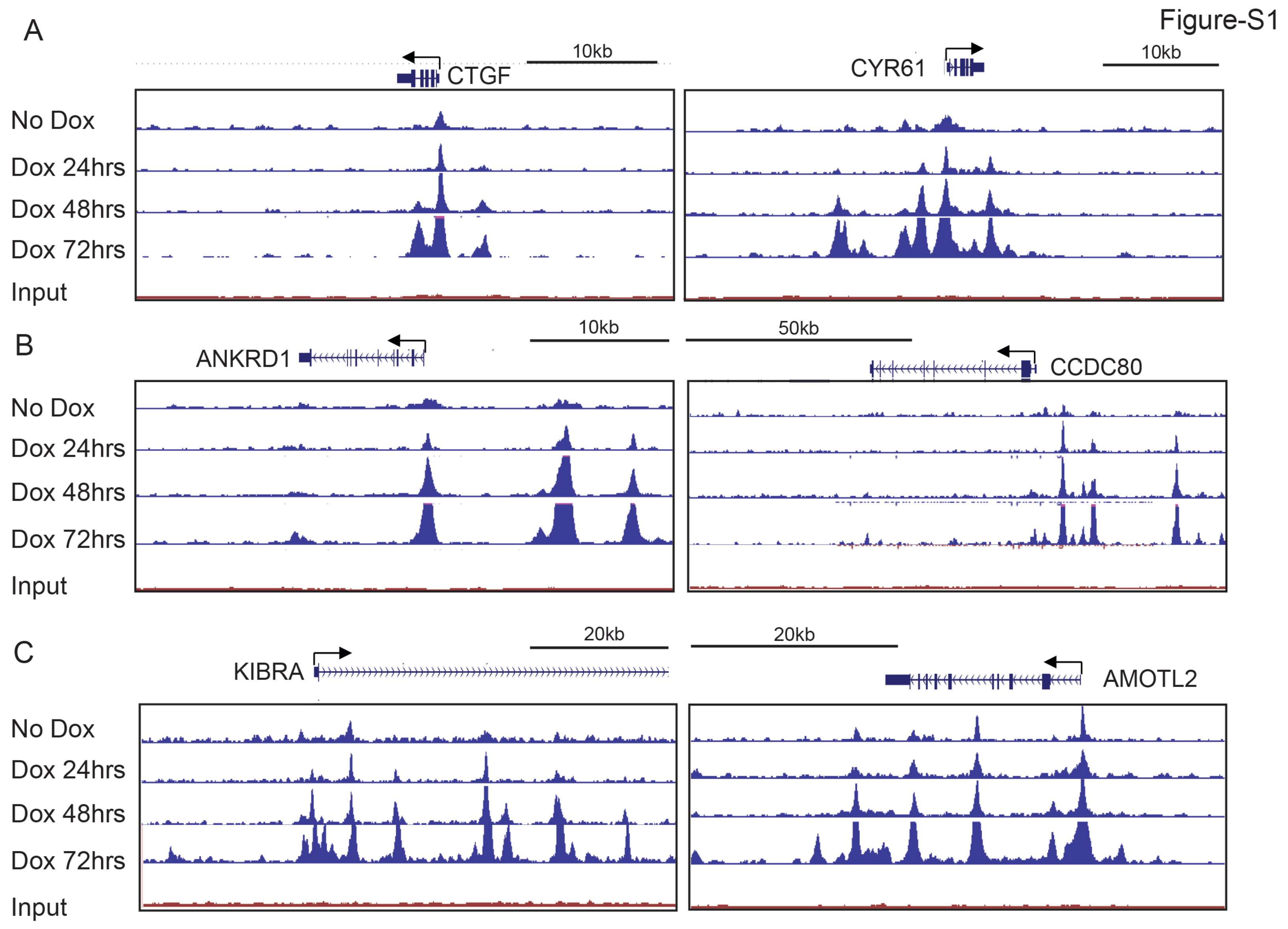
Figure-S1
A
10kb
CTGF
CYR61
10kb
No Dox
Dox 24hrs
Dox 48hrs
Dox 72hrs
Input
B
10kb
50kb
ANKRD1
CCDC80
No Dox
Dox 24hrs
Dox 48hrs
Dox 72hrs
Input
C
20kb
20kb
KIBRA
AMOTL2
No Dox
Dox 24hrs
Dox 48hrs
Dox 72hrs
Input

Figure-S2

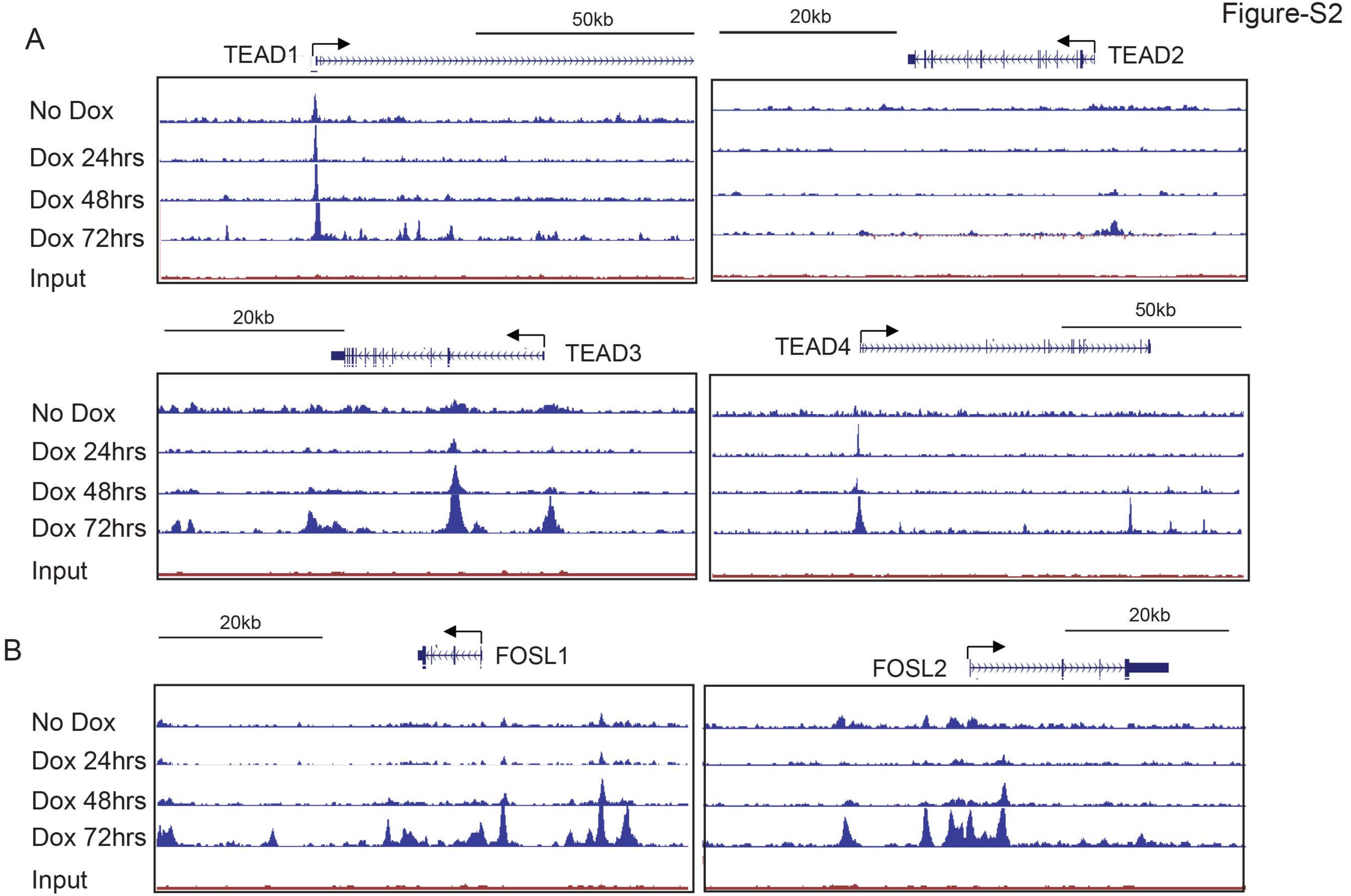

A
50kb
TEAD1
20kb
TEAD2
No Dox
Dox 24hrs
Dox 48hrs
Dox 72hrs
Input
20kb
TEAD3
50kb
TEAD4
No Dox
Dox 24hrs
Dox 48hrs
Dox 72hrs
Input
B
20kb
FOSL1
20kb
FOSL2
No Dox
Dox 24hrs
Dox 48hrs
Dox 72hrs
Input
