# Supplementary figures and images for: Genome-Wide Characterization of TAZ Binding Sites in Mammary Epithelial Cells
Source: Cancers (Basel). 2023 Sep 25;15(19):4713. doi: 10.3390/cancers15194713 (PMC10571831; doi:10.3390/cancers15194713)

# Original western blot

Figure 1A.

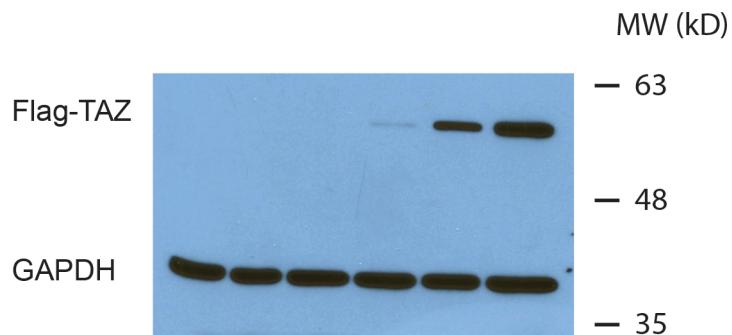

Figure 1B.

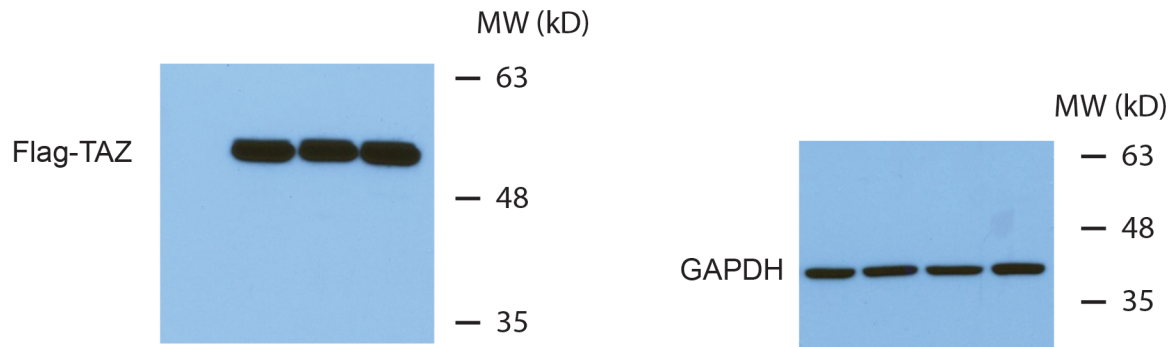

Supplement: Supplementary file 1 [file cancers-15-04713-s001.zip › cancers-2543387-original-images.pdf]
